# Exercise in allogeneic bone marrow transplantation: a qualitative representation of the patient perspective

**DOI:** 10.1007/s00520-022-06931-9

**Published:** 2022-03-16

**Authors:** Shaza Abo, Selina M. Parry, David Ritchie, Gabriella Sgro, Dominic Truong, Linda Denehy, Catherine L. Granger

**Affiliations:** 1 Department of Physiotherapy, The University of Melbourne, Parkville, VIC 3010 Australia; 2 Department of Physiotherapy, Royal Melbourne Hospital, Parkville, VIC 3052 Australia; 3 Clinical Haematology, Peter MacCallum Cancer Centre and Royal Melbourne Hospital, Parkville, VIC 3052 Australia; 4 Department of Allied Health, Peter MacCallum Cancer Centre, Melbourne, VIC 3000 Australia

**Keywords:** Allogeneic bone marrow transplantation, Stem cell transplantation, Exercise, Quality of life, Patient experience, Survivorship

## Abstract

**Purpose:**

Exercise is emerging as a vital aspect of care to alleviate the physical and psychosocial symptom burden associated with allogeneic bone marrow transplantation (BMT). Understanding the patient perspective regarding exercise is important to move towards implementation. This study aimed to characterise experiences and views regarding participation in an exercise program in adults receiving treatment for haematological disease with allogeneic BMT.

**Methods:**

Individual semi-structured interviews were conducted with 35 participants from either an early- or late-commencing supervised group-based exercise program. Using an inductive, conventional approach to qualitative content analysis data were independently analysed by two researchers.

**Results:**

Six major themes and 33 sub-themes were identified: this encompassed motivation, physical opportunity and capability to exercise; psychosocial effects of group-based exercise; experienced impact of participation in an exercise program; and intervention design considerations. Key barriers to exercise included symptom severity and fluctuating health and distance or difficult access to an exercise facility or equipment, whilst facilitators included encouragement from staff; peer support in the group-based setting; flexibility; education; and ability to measure change.

**Conclusion:**

This study highlights the importance of a flexible approach to exercise with consideration of individual symptoms and preferences. The perceived psychological impact of exercise should not be underestimated; future exercise programs should be designed in partnership with patients, with consideration of group-based activities to reduce social isolation if this is feasible in the treatment context. Intervention design should also acknowledge the individual’s physical and psychological capability, opportunity and automatic and reflective motivation to direct and sustain exercise behaviours following BMT.

**Supplementary Information:**

The online version contains supplementary material available at 10.1007/s00520-022-06931-9.

## Introduction

Allogeneic bone marrow transplantation (BMT) for people with haematological disease is an intensive medical treatment which can result in significant symptom burden[1–4]. Recipients experience fatigue, reduced exercise capacity, strength and health-related quality of life (HRQoL)[1, 3, 5, 6]. A recent meta-analysis of RCTs found that exercise can improve these symptoms, particularly in allogeneic BMT[7–11]; however, structured exercise is not part of standard care in most centres worldwide. The reasons for this limited access to exercise in BMT are likely multifactorial, and may include historical caution regarding safety of exercise during cytopenia[12], that RCTs to date have been of poor-moderate methodological quality and/or have relatively small sample size (median sample size of 42)[7]; there is an absence of consensus regarding ideal exercise intervention design (frequency, intensity, timing, type), and lack of research into patient exercise preferences. Furthermore, implementation science suggests that clinical uptake of new interventions relies upon identification of barriers and facilitators from key stakeholders, including patients[13].

There is a paucity of research examining the patient perspective regarding physical activity or exercise in BMT. Qualitative research in BMT has predominantly been in multiple myeloma and/or autologous BMT[14–17] and that in allogeneic BMT has focused on symptoms rather than participation in an exercise program[18, 19]. In allogeneic BMT, patients perceive physical incapacity and/or reduced self-efficacy as key issues post-transplant hence increased access to specialised exercise support is required[18, 19].

We recently conducted two prospective cohort studies to test the feasibility of group-based exercise at different time points during the continuum of allogeneic BMT[1, 5]. To extend our understanding of acceptability and feasibility to exercise participation in allogeneic BMT, we conducted a qualitative study alongside this work. This study aimed to explore the experiences and views of participants completing an early- and late-commencing supervised group-based exercise program during treatment for haematological disease with allogeneic BMT. The secondary aims were to characterise participant views regarding program timing, acceptability, expectations, barriers and facilitators.

## Methods

### Study design

The reporting of this study was guided by the consolidated criteria for reporting qualitative research guidelines [20], with further details regarding study methodology in Supplementary File 1.

#### Participant selection, sample size and setting

**Fig. 1:**
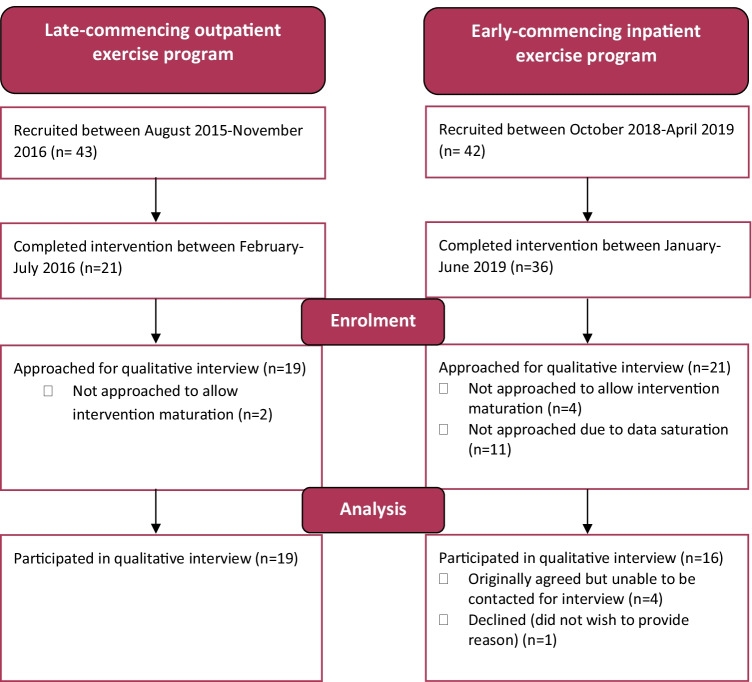
Participant recruitment from each intervention study to the qualitative study

Participants were recruited from two single-group cohort studies which tested the feasibility of an early-commencing inpatient group-based exercise program [5] and a late-commencing outpatient group-based exercise program [1]. Inclusion criteria in both studies were English-speaking adults able to provide written consent receiving treatment for a haematological disease with allogeneic BMT at a tertiary hospital in Melbourne, Australia. Ethical approval was obtained for both studies (HREC 2015.095 and 2018.053). Within 2 weeks following exercise program completion, consecutive participants were approached to conduct an interview. To allow for intervention maturation, participant recruitment for qualitative interviews commenced after approximately the first 10% of participants had completed the intervention. Figure 1 demonstrates participant recruitment and reasons for non-participation.

Table 1 provides a summary of the intervention in both the early- and late-commencing exercise studies and further details are published elsewhere [1, 5]. The interventions incorporated education in addition to mixed aerobic and resistance training in a group-based setting. The early-commencing program started upon hospital admission, whilst the late-commencing program started at approximately 60 days post-transplant.

**Table 1:** Summary of the two exercise programs

Time	Early-commencing inpatient exercise program[5]	Late-commencing outpatient exercise program[1]
Pre-transplant	Assessment with physiotherapist + physical activity advice	Assessment with physiotherapist + physical activity advice
During inpatient hospital stay for transplant	Supervised^a^ on BMT unit of hospital: 1 h, 5 days/week Unsupervised: 2 days/week	Nil
Hospital discharge to day 60 post-transplant	Unsupervised home-based exercise program, goal to complete 3–5 days/week; 1 day/week phone call^a^ to encourage + progress exercise as needed	Nil
Day 60 to approximately day 100 post-transplant	Study ceased at day 60; participants continued with routine care which by this stage included an outpatient exercise program	Supervised^a^: in outpatient hospital gymnasium for 1 h, 1 day/week Unsupervised: home-based program, goal to complete 3–5 days/week
Type and intensity of exercise	Group-based; mixed aerobic and resistance; individualised at low-moderate intensity	Group-based; mixed aerobic and resistance; individualised at moderate intensity
Exercise “tools”	Exercise diary × 2: (1) to complete during inpatient admission; (2) to complete from discharge to 60 days post-transplant	Provision of “FitBit” activity tracker and exercise diary to complete from day 60 to day 100 post-transplant

^a^Supervised exercise sessions and follow-up phone calls were led by a physiotherapist with expertise in oncology care

### Data collection and research team reflexivity

Participant demographics, including baseline physical activity levels, and feasibility data, including percentage attendance at available sessions, were collected. A semi-structured interview schedule (Supplementary File 2) was developed to gather participant feedback regarding the intervention, in particular acceptability, program expectations, prior experiences and beliefs regarding timing, barriers and facilitators.

Interviews were conducted one-on-one with one of five physiotherapy staff (two male, three female) who had experience working with people recovering from cancer treatment. Two physiotherapists (one male (Masters degree), one female (PhD)) had experience in qualitative research, one of which (male) conducted initial interviews which were used as part of training for the other interviewers. The majority of interviews were conducted by a female physiotherapist (Bachelor degree, GS, *n* = 16 interviews) and a male allied health assistant (Bachelor degree in exercise physiology, *n* = 13 interviews). To check for consistency and as part of training, the study lead (SA) also reviewed the first 2 interview recordings from these interviewers and verbal prompts were added to maximise depth. The interviewers had no prior contact with participants and were not biased by research interests in this topic, though may have faced time constraints due to actively working as clinicians in a busy tertiary hospital and scheduling of interviews during clinical hours.

Interviews were conducted a maximum of 4 weeks following program completion. A pragmatic target sample size of 15–20 participants from each program was chosen [21, 22]. Given interviews were relatively short in length (average length 7.2 min, range 3.1 to 15 min), to facilitate sufficiently rich data to answer the research aim, data from both the outpatient and inpatient studies were combined rather than analysed separately.

### Data analysis

Interviews were recorded and transcribed verbatim (GS), then checked by a second independent researcher for accuracy (SA). Non-identifiable transcripts were uploaded into NVivo software (released in March 2020; QSR International Pty Ltd.) and independently coded by two researchers (SA, SMP). Data from the two cohorts were analysed together, with transcripts identified as pertaining to participants from the outpatient or inpatient program using “OP” or “IP”, respectively. Participant review of transcripts was deemed inappropriate due to risk of mortality in this population and time elapsed from interview to analysis (2–5 years) [21, 23].

An inductive, conventional approach to qualitative content analysis as described by Hsieh and Shannon (2005) was used to analyse the data [24, 25]. Each transcript was read from start to finish, and then line by line to derive codes that appeared to capture participants’ perspectives [24]. The researchers independently grouped initial codes into categories to develop major themes and sub-themes from the data [24]. One researcher (SA) led both inpatient and outpatient interventions hence was innately engaged in the research topic and with the participants. Therefore, the independent researchers met after coding the first five interviews to check that preliminary interpretation of data into codes appropriately represented participant perspectives and maximise credibility within a naturalistic paradigm[24, 25]. The two researchers cross-checked the categorisation of codes into final themes and sub-themes until consensus was reached [24]. A third researcher (CG) reviewed the final themes alongside extracts from interview transcripts to check that the emerging themes were representative of participant quotations.

## Results

A total of 35 participants completed qualitative interviews, 19 from the late-commencing outpatient exercise program and 16 from the early-commencing inpatient exercise program. Thirty-two interviews were conducted face-to-face, and 3 via telephone.

### Participant characteristics

Table 2 provides a summary of participant demographics for the inpatient and outpatient exercise programs, including study participants who did not provide interview data. Participants were predominantly male (62.5% and 52.6%), with a mean (SD) age of 46.9 (12.6) and 44.0 (13.4) in the inpatient and outpatient programs respectively. The highest number of participants received their allogeneic BMT for acute myeloid leukaemia and most participants were meeting physical activity guidelines pre-transplant. There were no significant differences in those who did or did not provide qualitative data with regard to baseline demographics or attendance to the exercise sessions, although it is worth noting that the outpatient exercise program had significant drop-out due to death or deterioration in health status [1].

**Table 2:** Demographics of participants from the inpatient and outpatient studies who did or did not provide qualitative data

	Participants of the early-commencing inpatient study		Participants of the late-commencing outpatient study	
	Provided qualitative data (*n* = 16)	Did not provide qualitative data (*n* = 26^a^)	Provided qualitative data (*n* = 19^a^)	Did not provide qualitative data (*n* = 24^a^)
Age at baseline, mean (SD) (years)	46.9 (12.6), range 24–64	52.9 (14.0), range 27–72	44.0 (13.4), range 21–65	46.4 (15.2), range 18–68
Sex, male (%)	10 (62.5)	19 (73.1)	10 (52.6)	16 (66.7)
BMI (kg/m^2^)	27.2 (5.9)	27.3 (4.0)	26.2 (5.5)	26.3 (4.7)
Diagnosis, *n* (%)				
- AML	5 (31.3)	10 (38.5)	11 (57.9)	13 (54.2)
- B-ALL	2 (12.5)	6 (23.1)	1 (5.3)	2 (8.3)
- MM	3 (18.8)	1 (3.8)	4 (21.1)	0
- ALL	2 (12.5)	2 (7.7)	0	2 (8.3)
- T-ALL	3 (18.8)	2 (7.7)	0	1 (4.2)
- MDS	0	1 (3.8)	1 (5.3)	3 (12.5)
- T-Cell LGLL	1 (6.3)	2 (7.7)	1 (5.3)	0
- AA	0	1 (3.8)	0	2 (8.3)
- CML	0	1 (3.8)	0	1 (4.2)
Allogeneic transplant type				*n* = *20*
- Sibling or related donor	6 (37.5)	6 (23.1)	9 (47.4)	6 (30)
- MUD	6 (37.5)	13 (50)	9 (47.4)	14 (70)
- Cord	1 (6.3)	0	1 (5.3)	0
- Haploidentical	3 (18.8)	7 (26.9)	0	0
Meeting physical activity guidelines pre-transplant, *n* (%)	12 (75)	16 (64), *from n* = *25*	12 (70.6), *from n* = *17*	15 (68.2), *from n* = *22*
Attendance at group-based exercise program (%)^b^	62%, from *n* = 14	55%, from *n* = 21	72%, from *n* = 19	57%, from *n* = 7

^a^Except where specified that sample was lower due to missing data

^b^Attendance is reported as a percentage of sessions attended out of total scheduled sessions

Abbreviations: *AA*, aplastic anaemia;* ALL*, acute lymphoblastic leukaemia; *AML*, acute myeloid leukaemia; *B-ALL*, B-cell acute lymphoblastic leukaemia; *CML*, chronic myeloid leukaemia ; *BMI*, body mass index; *MDS*, myelodysplastic syndrome; *MM*, multiple myeloma; *MUD*, matched unrelated donor; *T-ALL*, T-cell acute lymphoblastic leukaemia; *T-Cell LGLL*, T-cell large granular lymphocytic leukaemia

### Views and experiences of exercise program participation

**Fig. 2:**
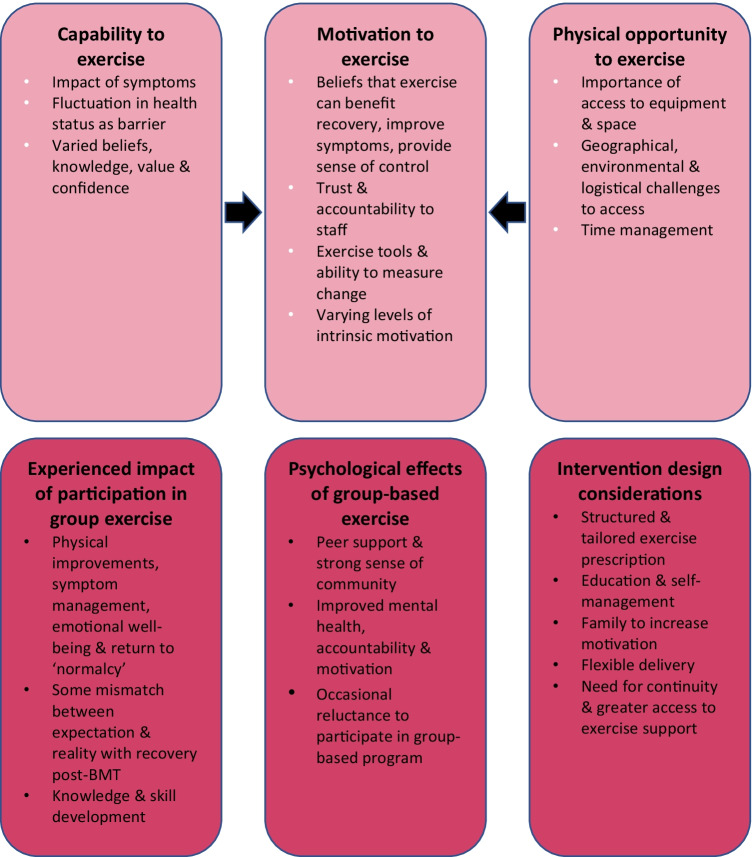
Summary of the major themes and sub-themes developed from participant perspectives. Three of the six major themes linked strongly to the COM-B model [26]

Six major themes and 33 sub-themes were derived from the data. Three of the major themes aligned with the COM-B behavioural change model [26] incorporating capability, opportunity and motivation to exercise. Three major themes related to the psychosocial effects of and experienced impact of group-based exercise, and intervention design considerations. Overall, the data elucidated that participants desire a flexible and individualised approach with consideration of the COM-B domains of capability, opportunity and motivation to exercise during and after allogenic BMT [26]. Figure 2 provides a summary and Supplementary File 3 provides additional participant quotations to support each theme.

#### Major theme 1: Motivation, automatic or reflective, to exercise during or after BMT

Participants obtained reflective motivation from belief that exercise would benefit recovery from transplant, and more specifically improve physical symptoms of treatment. For some, this belief stemmed from impact of previous hospitalisation. Participants perceived exercise would provide a sense of control, structure, or distraction from medical treatment during transplant.

- IP06: “…when I’ve previously been in hospital, I’ve realized that my muscle mass can waste pretty quickly so I was keen not to let that happen again.”
- OP20: “…it was something to keep the motivation in there and try and maintain a certain level of fitness and give me something to look forward to throughout treatment as well.”

Automatic motivation came from a sense of accountability to staff with whom participants built a relationship based on trust in their expertise. Particularly when feeling unwell or self-motivation was low, participants sought inspiration to exercise from the multidisciplinary team or through peer support in a group setting. Participants found exercise tools such as the diary or “FitBit” step-counting device provided behavioural regulation to motivate them to exercise. An ability to measure change through formal or informal outcomes also provided incentive.

- IP12: “…it would make me accountable… particularly when I was on the ward…feeling terrible, you know that [physiotherapy staff] were there to get you out of bed and to your session, and that just helps you maintain that momentum…”.
- OP41: “…the little counter encourages you to go out and buy the paper or go for another walk, even when the weather is lousy…”.

Often associated with prior exercise habits, some participants discussed strong internal motivation to exercise. Others reported a lack of intrinsic motivation, particularly with self-directed aspects of the exercise programs.

- IP22: “I’m a very active person…a very self-motivated person so that would help me…”.
- OP14: “Barrier-wise, was probably my own self-motivation…other than coming in here…convincing yourself that you’ve got to get out and do it…”.

#### Major theme 2: Physical opportunity to exercise

Access to equipment and a suitable therapy space across the supervised inpatient, outpatient and unsupervised home-based setting was considered important to facilitate exercise participation. In the outpatient and home-based setting, geographical, environmental, weather and logistical challenges were raised. Participants reported that having easy access to or living close to an exercise facility aided participation, whilst distance or difficult access such as parking were perceived as barriers. Some participants raised inclement weather as a challenge to conducting self-directed exercise such as walking outdoors. For some participants, managing time for exercise was difficult within a busy personal lifestyle, and for others, the challenge was coordinating time around their busy hospital appointment schedule.

- OP39: “…in winter, if I didn’t have a treadmill you just don’t want to go out in the cold, and you don’t want to catch anything if you’re neutropenic.”
- IP05: “…you’ve got a lot more going on at home, so it is a bit harder to fit it in.”

#### Major theme 3: Capability to exercise

Participants raised several factors that impacted on physical, and, to a lesser degree, psychological capability to exercise. Treatment-related side effects and symptom burden were viewed as the main barrier to participation in an exercise program. Severity of symptoms in some cases was debilitating, especially if requiring admission to intensive care, and was particularly seen as a barrier to exercise in the inpatient program. Fatigue and/or weakness were commonly reported as limiting participation across the transplant continuum. Fluctuation in health status was often considered a barrier to physical capability as varying, unpredictable symptoms would dictate ability to exercise.

- IP09: “I had good and bad days… Some days I get really so fatigued that all I want to do is sleep, and my bones hurt so bad that I can’t even exercise. And it’s extremely frustrating.”
- OP39: “…fatigue and not feeling well and me feeling nauseous and everything, and I just thought I don’t think I can do this.”

Beliefs, knowledge, value and confidence to exercise varied among participants and played a role in psychological capability to exercise. Some participants felt confident in their knowledge that exercise would support recovery which facilitated involvement; others were apprehensive that they were not physically or mentally ready to exercise.

#### Major theme 4: Psychosocial effects of group-based exercise

Most participants highlighted a feeling of peer support and strong sense of community or accountability obtained from exercising alongside others going through a shared experience. Some noted that seeing others exercise despite feeling unwell provided perspective; similarly, seeing others who were ahead in their transplant trajectory provided motivation. Exercise, particularly through the social interaction garnered in a group setting, was seen as supporting mental health. Participants raised that the healthcare environment can be isolating or dehumanising and attending a group-based program helped to mitigate this.

IP26: “I think it provides such a good opportunity…to meet people and talk to people and see where everyone else is at and see that you actually are still a person…It takes it away from the medical stuff and you just get to be you and exercise…that’s really important mentally and physically.”

Two participants from the inpatient program raised reluctance toward group-based exercise. One reported preferring an individual approach due to concerns that emotional investment in others would negatively impact individual progress or psychological state, and another voiced concerns about infection risk.

#### Major theme 5: Experienced impact of participation in an exercise program

There was a unanimous perception that participation in an exercise program was beneficial. Participants experienced physical improvements in strength and/or symptom management, particularly fatigue or energy levels. Some participants expressed surprise that experienced benefit was greater than anticipated whilst some reported a mismatch between their expectations and reality, often attributed to unexpected treatment-related side effects or lack of motivation that limited exercise participation and overall recovery.

- IP05: “I didn’t feel like I came out really frail, or that I’d dropped my level of fitness, I felt like I had maintained it quite well which surprised me, I thought I’d be in a far worse state when I got out of hospital. I hadn’t given probably the mental well-being side much thought, but it really helped with that as well.”
- OP43: “…because I had so many issues down the way, I don’t feel like I am where I was hoping to be…just me putting too much pressure on myself and expecting too much.”

Many participants experienced benefits to emotional well-being or self-confidence, often associated with a feeling of improved physical health or sense of accomplishment through exercise. Returning to “normal” life or pre-transplant self was important and it was perceived that participation in exercise facilitated this. The program was perceived to have led to knowledge attainment and skill development to implement exercise as part of a healthy lifestyle.

- OP14: “…to actually get yourself up and going and it gives you a bit of personal pride and a bit of get up and go…it’s a fantastic program from a mental point of view and a physical point of view.”
- IP08: “…I learn the importance of exercise in our daily lives.”

#### Major theme 6: Intervention design considerations

There were a number of suggestions to improve design or maximise participation in future exercise programs. Participants highlighted the importance of tailored exercise prescription based on symptoms, comorbidities, age and interests given the broad range of people receiving BMT. Participants felt that education was important to incentivise or empower self-management. Some perceived incorporation of nutrition advice to be particularly important.

- OP34: “…as long as it’s tailored to the person, their limitations cause there’s all sorts of ages and health concerns and whatever, that’s the main thing…”
- IP15: “It has to be explained that it is crucial for them to…exercise.”

Views regarding ideal timing of an exercise program across the transplant continuum varied. Some perceived the commencement time was just right. Participants from the inpatient setting generally expressed a desire to start exercise as early as possible, before treatment-related side effects and/or symptoms limited participation. A minority pondered whether delaying commencement until symptoms abated would be better. Participants in the outpatient setting felt that the program could have started earlier, and some suggested it should commence in the pre-transplant phase to maximise physical strength and fitness.

There was an overall desire for greater access to exercise support across the transplant continuum. Inpatients felt that following hospital discharge, there was inadequate support to maintain exercise habits developed during their admission and would have liked to continue to attend a group-based program. Outpatients felt that the supervised program once per week was too infrequent to maintain motivation for self-directed exercise.

- OP39: “I actually think that it should be done leading up to the bone marrow transplant...to be a bit stronger and fitter leading into the transplant.”
- IP05: “I’d design the program so that it went from the start of your time in hospital through to day-100...”

Flexibility around scheduling of program delivery throughout the day from an inpatient perspective and around medical appointments from an outpatient perspective was considered important to facilitate exercise participation. One participant suggested that involvement of family may increase motivation to exercise. Whilst participants acknowledge some challenges, overwhelmingly it was felt that structured exercise should be embedded as an essential aspect of routine recovery from transplant.

- OP34: “So, it’s a no-brainer to have some sort of structured program to help people, as they come out of those beds to do something, otherwise they’ll be lying on a bed for three months at home, and they’ll be nowhere in 100 days…”

## Discussion

To our knowledge, this is the first qualitative study to ascertain views and experiences of exercise among adults undergoing allogeneic BMT, which is vital to guide future research and implementation of exercise interventions. The key themes derived help to understand sources of motivation, barriers and facilitators to opportunity and capability to exercise, the psychological effects of group-based exercise and experienced impact of participation in an exercise program. Given the complexities associated with allogeneic BMT, clinicians can use this study to gain insights regarding patient preferences and views to support implementation of exercise in practice. This study adds to the literature that places the individual at the centre of the healthcare experience and reminds us of the importance of person-centred care in design and application of future interventions.

Experiences of participants in our study link strongly with the “COM-B” behavioural change model which suggests that capability, opportunity and motivation all contribute towards enacting a behaviour [26]. The COM-B model can be used in line with results of this study to design sustainable future interventions [26]. For example, similar to other research, our study identified that symptoms, especially fatigue and/or weakness, and fluctuating health status can impact capability to exercise [15]. Whilst the significant and well-documented symptom burden of BMT [2, 3, 6, 27, 28] may be difficult to alter, the lived experience of symptoms on patients’ ability to exercise should be acknowledged and exercise should be flexible to accommodate fluctuations in health status. Furthermore, participants in our study identified that improving knowledge of the importance of exercise on recovery could be used to incentivise exercise; thus, education should be incorporated to maximise psychological capability. Physical and social opportunity to exercise can be maximised through environmental change [26]; our study suggests flexible structuring of exercise around medical appointments and, on par with Parisek and colleagues (2021), ensuring consistent provision of exercise support from pre- to post-transplant [18]. Regarding motivation to exercise in BMT, our study identified that support from staff, exercise tools and outcome measurement of physical change provided automatic incentive, whilst belief that exercise would provide a sense of control or benefit recovery provided reflective motivation. This flexible, individualised COM-B approach also aligns with the recommendation that oncology clinicians “ask, assess, refer” in order to facilitate exercise adoption into routine care during and following cancer treatment worldwide [29].

Understanding our results within the international healthcare context is important. Our study was conducted in a tertiary hospital in Melbourne, Australia, where the benefits of exercise in BMT are well-understood by the treating team including allied health, physicians and nurses. Evidence in breast cancer and chronic disease demonstrates that exercise advice from a medical professional can improve physical activity levels and subsequently health outcomes[30, 31]. Whilst our participants perceived their clinical team as a source of motivation to exercise, a recent qualitative study in people treated with autologous BMT who completed an inpatient exercise program in Korea reported a barrier to exercise was lack of encouragement from the oncologist [17]. A recent UK-based survey found that health professionals potentially lack confidence and/or knowledge around provision of physical activity advice in haemato-oncology [32]. Our results perhaps align more closely with qualitative studies in Europe which demonstrate that people with haematological malignancies feel overwhelmed by the diagnosis and treatment decision-making process and participation in an exercise program provided an active role to psychologically and physically reduce symptom burden during BMT [33, 34]. Another qualitative study conducted in patients with multiple myeloma in Australia perceived psychological benefits of physical activity to be even greater than physical which was also attributed to a feeling of accomplishment or helping to cope with disease [15]. A recent international “call to action” advised future research regarding exercise in oncology to focus on implementation science and health services research [29]. Whilst our study provides the patient perspective, one important element of implementation science, design of future interventions, should consider multiple levels of context, including clinician and organisation perspectives and cost-effectiveness to maximise sustainability [13].

Whilst there is growing research to support the benefits of exercise on HRQoL, including emotional well-being, in BMT recipients [7, 35, 36], no RCTs have explored the efficacy of group-based exercise. Group-based exercise may offer benefits of peer support, camaraderie and help to maintain motivation. Group-based exercise rehabilitation in other health conditions such as chronic pulmonary or cardiac disease has demonstrated improvements in psychological health [37]. Whilst the research regarding the effects of group-based peer support on psychological outcomes in general cancer is still unclear [38], group-based exercise appears to improve engagement in physical activity in the breast cancer cohort [39]. In BMT, recent qualitative studies report participant feelings of anxiety associated with isolation during transplant [17]; and that the effects of a BMT, particularly social isolation, fatigue and reduced physical capabilities, contribute to low mood and subsequently reduced motivation to exercise [19]. Our participants perceived that exercise, and largely the group-based approach, helped to alleviate these feelings. Beyond adding to existing literature that patients prefer supervised over self-directed exercise which in itself may offer emotional support from healthcare professionals [17], the novel group-based approach to exercise in BMT was experienced as an unexpected highlight by most participants due to perceived benefits to psychological well-being. This perception is supported by the quantitative finding that emotional well-being remained stable and significantly improved in the late- and early-commencing exercise programs respectively [1, 5].

## Study strengths and limitations

Our study provides valuable insights to guide design of future exercise interventions in this population. There are a number of critical limitations which need to be acknowledged. Firstly, our sample may represent participants who found the intervention more acceptable or derived the greatest benefits given only participants who completed the intervention were approached; future studies should explore avenues to gather qualitative data from withdrawn participants or those with varying levels of exercise compliance. Secondly, the interviews were of short duration (7.2 min average). However, we involved a relatively large sample size for this type of study (35 participants). Thirdly, this study was conducted in a single major hospital in one country and involved no member checking of the data by the participants involved in the interview process. Therefore, caution is required with interpretation of the findings of our qualitative study. Further in-depth qualitative studies are required to confirm the findings of our study.

This study is strengthened by the following of guidelines for conducting qualitative studies including the use of duplicate transcription and two independent researchers to conduct data analysis. This is the first qualitative study of exercise experiences in allogeneic BMT and the first to explore the value of group-based exercise in BMT. Further strengths include that data were collected in a pragmatic hospital setting which increases generalisability.

## Conclusion

This qualitative study highlights the importance of an individualised, flexible approach to exercise prescription in people treated for haematological disease with allogeneic BMT. Structured and/or group-based exercise with emphasis on education was perceived as a counterbalance to the experienced social isolation and disempowerment during BMT. The perceived psychological impact of exercise should not be underestimated; future exercise programs should be designed in partnership with patients, with consideration of group-based activities to reduce social isolation if this is feasible in the treatment context. Intervention design should also consider aligning with implementation science and behaviour change systems to acknowledge the individual’s physical and psychological capability, opportunity and automatic and reflective motivation to direct and sustain exercise behaviours following BMT.

## Supplementary Information

Below is the link to the electronic supplementary material.

Supplementary file1 (DOCX 35.3 kb)

## Data Availability

Not applicable.

## References

[1] Abo S, Granger CL, Denehy L, Ritchie D, Panek-Hudson Y, Irving L (2018). A hospital and home-based exercise program to address functional decline in people following allogeneic stem cell transplantation. Support Care Cancer.

[2] Baker KS, Ness KK, Weisdorf D (2010). Late effects in survivors of acute leukemia treated with hematopoietic cell transplantation: a report from the bone marrow transplant survivor study. Leukemia.

[3] Dirou S, Chambellan A, Chevallier P (2018). Deconditioning, fatigue and impaired quality of life in long-term survivors after allogeneic hematopoietic stem cell transplantation. Bone Marrow Transplant.

[4] Wingard JR, Huang IC, Sobocinski KA (2010). Factors associated with self-reported physical and mental health after hematopoietic cell transplantation. Biol Blood Marrow Transplant.

[5] Abo S, Ritchie D, Denehy L, Panek-Hudson Y, Irving L, Granger CL (2021). Feasibility of early-commencing group-based exercise in allogeneic bone marrow transplantation: the BOOST study. Bone Marrow Transplant.

[6] Ishikawa A, Otaka Y, Kamisako M (2019). Factors affecting lower limb muscle strength and cardiopulmonary fitness after allogeneic hematopoietic stem cell transplantation. Support Care Cancer.

[7] Abo S, Denehy L, Ritchie D et al. (2021) People with hematological malignancies treated with bone marrow transplantation have improved function, quality of life and fatigue following exercise intervention: a systematic review and meta-analysis. Phys Ther; 101(8):pzab130. doi:10.1093/ptj/pzab130 10.1093/ptj/pzab130 33989413

[8] Hacker ED, Collins E, Park C, Peters T, Patel P, Rondelli D (2017). Strength training to enhance early recovery after hematopoietic stem cell transplantation. Biol Blood Marrow Transplant.

[9] Wiskemann J, Dreger P, Schwerdtfeger R (2011). Effects of a partly self-administered exercise program before, during, and after allogeneic stem cell transplantation. Blood.

[10] Knols RH, de Bruin ED, Uebelhart D (2011). (2011) Effects of an outpatient physical exercise program on hematopoietic stem-cell transplantation recipients: a randomized clinical trial. Bone Marrow Transplant.

[11] Baumann FT, Kraut L, Schule K, Bloch W, Fauser AA (2010). (2010) A controlled randomized study examining the effects of exercise therapy on patients undergoing haematopoietic stem cell transplantation. Bone Marrow Transplant.

[12] Elter T, Stipanov M, Heuser E (2009). Is physical exercise possible in patients with critical cytopenia undergoing intensive chemotherapy for acute leukaemia or aggressive lymphoma?. Int J Hematol.

[13] Bauer MS, Kirchner J (2020). Implementation science: what is it and why should I care?. Psychiatry Res.

[14] Craike M, Hose K, Courneya KS, Harrison SJ, Livingston PM (2017). Physical activity preferences for people living with multiple myeloma: a qualitative study. Cancer Nurs.

[15] Craike MJ, Hose K, Courneya KS, Harrison SJ, Livingston PM (2013). Perceived benefits and barriers to exercise for recently treated patients with multiple myeloma: a qualitative study. BMC Cancer.

[16] Nicol JL, Woodrow C, Burton NW et al. (2020) Physical activity in people with multiple myeloma: associated factors and exercise program preferences. J Clin Med; 9(10). 10.3390/jcm9103277 10.3390/jcm9103277 PMC7601964 33066153

[17] Yu MS, An KY, Byeon J (2020). Exercise barriers and facilitators during hematopoietic stem cell transplantation: a qualitative study. BMJ Open.

[18] Parisek M, Loss J, Holler E et al. (2021) “This graft-vs.-host disease determines my life. That’s it.”—a qualitative analysis of the experiences and needs of allogenic hematopoietic stem cells transplantation survivors in Germany. Frontiers in Public Health. 2021;9(785). 10.3389/fpubh.2021.687675 10.3389/fpubh.2021.687675 PMC8280766 34277549

[19] Freeman AT, Stover AM, Grover NS, Shea TC, Reeve BB, Wood WA (2017). Patient perspectives on physical function after allogeneic hematopoietic stem cell transplantation: a qualitative study. Bone Marrow Transplant.

[20] Tong A, Sainsbury P, Craig J (2007). Consolidated criteria for reporting qualitative research (COREQ): a 32-item checklist for interviews and focus groups. Int J Qual Health Care.

[21] Varpio L, Ajjawi R, Monrouxe LV, O’Brien BC, Rees CE (2017). Shedding the cobra effect: problematising thematic emergence, triangulation, saturation and member checking. Med Educ.

[22] Braun V, Clarke V (2021). To saturate or not to saturate? Questioning data saturation as a useful concept for thematic analysis and sample-size rationales. Qual Res Sport Exerc.

[23] Birt L, Scott S, Cavers D, Campbell C, Walter F (2016). Member checking: a tool to enhance trustworthiness or merely a nod to validation?. Qual Health Res.

[24] Hsieh HF (2005). Shannon SE (2005) Three approaches to qualitative content analysis. Qual Health Res.

[25] Liamputtong P (2020). Qualitative research methods 5e EBook., Melbourne, AUSTRALIA: Oxford University Press Australia & New Zealand.

[26] Michie S, van Stralen MM, West R (2011). The behaviour change wheel: a new method for characterising and designing behaviour change interventions. Implementation Sci.

[27] Morishita S, Kaida K, Ikegame K (2012). Impaired physiological function and health-related QOL in patients before hematopoietic stem-cell transplantation. Support Care Cancer.

[28] Garcia CM, Mumby PB, Thilges S, Stiff PJ (2012). Comparison of early quality of life outcomes in autologous and allogeneic transplant patients. Bone Marrow Transplant.

[29] Schmitz KH, Campbell AM, Stuiver MM (2019). Exercise is medicine in oncology: engaging clinicians to help patients move through cancer. CA Cancer J Clin.

[30] Jones LW, Courneya KS, Fairey AS, Mackey JR (2004). Effects of an oncologist’s recommendation to exercise on self-reported exercise behavior in newly diagnosed breast cancer survivors: a single-blind, randomized controlled trial. Ann Behav Med.

[31] Berra K, Rippe J, Manson JE (2015). Making physical activity counseling a priority in clinical practice: the time for action is now. JAMA.

[32] McCourt O, Yong K, Ramdharry G, Fisher A (2021). Physical activity during and after haematological cancer treatment: a cross-sectional survey of haematology healthcare professionals in the United Kingdom. J Multidiscip Healthc.

[33] Ernst J, Berger S, Weißflog G (2013). Patient participation in the medical decision-making process in haemato-oncology–a qualitative study. Eur J Cancer Care (Engl).

[34] Jarden M, Holder J, Hovgaard D, Adamsen L (2014). The active patient (TAP): a qualitative study of the patients’ experience and appraisal of a multimodal rehabilitation intervention during hematopoietic stem cell transplantation [abstract]. Psycho oncology.

[35] Kim DS, Kim SH (2005). Effects of a relaxation breathing exercise on anxiety, depression, and leukocyte in hemopoietic stem cell transplantation patients. Cancer Nurs.

[36] Yildiz Kabak V, Goker H, Duger T (2020). Effects of partly supervised and home-based exercise program in patients undergoing hematopoietic stem cell transplantation: a case-control study. Support Care Cancer.

[37] McCarthy B, Casey D, Devane D, Murphy K, Murphy E, Lacasse Y (2015) Pulmonary rehabilitation for chronic obstructive pulmonary disease. Cochrane Database Syst Rev.;(2):CD003793. doi:10.1002/14651858.CD003793.pub3 10.1002/14651858.CD003793.pub3 PMC10008021 25705944

[38] Hoey LM, Ieropoli SC, White VM, Jefford M (2008). Systematic review of peer-support programs for people with cancer. Patient Educ Couns.

[39] Wurz A, St-Aubin A, Brunet J (2015). Breast cancer survivors’ barriers and motives for participating in a group-based physical activity program offered in the community. Supp Care Cancer.

