# Supplementary material for: Exercise in allogeneic bone marrow transplantation: a qualitative representation of the patient perspective
Source: Support Care Cancer. 2022 Mar 16;30(6):5389–99. doi: 10.1007/s00520-022-06931-9 (PMC9046308; doi:10.1007/s00520-022-06931-9)
Supplement: Supplementary file 1 — Supplementary file1 (DOCX 35.3 kb) [file 520_2022_6931_MOESM1_ESM.docx]

**Title:** Exercise in allogeneic bone marrow transplantation: a qualitative representation of the patient perspective

**Authors:** Shaza Abo^1,2^, Selina M Parry^1^, David Ritchie^3^, Gabriella Sgro^2^, Dominic Truong^1^, Linda Denehy^1,4^*, Catherine L Granger^1,2^*

*denotes joint senior author

**Affiliations:**

1. Department of Physiotherapy, The University of Melbourne, VIC 3010, Australia;
2. Department of Physiotherapy, Royal Melbourne Hospital, Parkville, VIC 3052, Australia;
3. Clinical Haematology, Peter MacCallum Cancer Centre and Royal Melbourne Hospital, Parkville, VIC 3052, Australia;
4. Department of Allied Health, Peter MacCallum Cancer Centre, Melbourne, VIC 3000, Australia.

**Corresponding author contact details:**

Ms Shaza S Abo

Department of Physiotherapy, The University of Melbourne, Victoria, 3010, Australia

Email: shaza.abo@unimelb.edu.au | ORCID 0000-0001-8383-9837

**Supplementary material**

**Supplementary File 1:** Further detail regarding study methodology as per the consolidated criteria for reporting qualitative research (COREQ) guidelines[20].

**METHODS**

***Research team and reflexivity***

Interviews were conducted by five staff members of the physiotherapy department, all of whom had experience working with people recovering from cancer treatment. The first 3 interviews were conducted by a male grade 3 (senior) clinician physiotherapist who had experience in qualitative research (Masters degree). The recordings of these first 3 interviews were used alongside interview schedule training by study lead who had conducted a course in qualitative research (SA) to train the further researchers. One interview was conducted by a female physiotherapist conducting separate post-doctoral research within the department (PhD); two interviews were conducted by a female grade 1 physiotherapist (Bachelor degree). The majority of interviews were conducted by a female grade 1 physiotherapist (Bachelor degree, GS, n=16) and a male allied health assistant (Bachelor degree as an exercise physiologist; n=13). To check for consistency and quality, the first 2 interview recordings from each interviewer were reviewed by study lead (SA). Participants knew that the interviewers were physiotherapy staff members who worked with the lead researcher (SA) but did not have particular interests in the research topic. All participants were known to the study lead through participation in the intervention also led by this researcher (SA). The interviewers had no prior contact with participants and were not biased by research interests in this topic, though may have faced time constraints due to actively working as clinicians in a busy tertiary hospital and scheduling of interviews during clinical hours.

The researchers who independently conducted the data coding and analysis were one female PhD-qualified academic physiotherapist (SMP) with 8 years of experience in qualitative research and one female physiotherapist PhD-candidate (SA) who had conducted formal training in qualitative research and was provided with mentorship from the other researcher involved in analysis (SMP) and senior authors (CG, LD). Given that one of the researchers involved in analysis led this study and the two group-based exercise studies as part of her PhD research (SA), we recognize that these factors may have influenced interpretation of data.

***Study design***

*Theoretical framework*

Conventional qualitative content analysis was chosen as the methodological orientation to underpin this study and data were analysed inductively as the existing literature in allogeneic BMT is still evolving and the aim was to avoid preconceived assumptions or categories[24, 25].

*Participant selection, sample size and setting*

Participants were recruited from two single-group cohort studies which tested the feasibility of an early-commencing inpatient group-based exercise program[5] and a late-commencing outpatient group-based exercise program[1]. Inclusion criteria for participation in both studies were English-speaking adults receiving treatment for a haematological disease with allogeneic BMT at a tertiary hospital in Melbourne, Australia. Ethical approval was obtained for both studies (HREC 2015.095 and 2018.053). Written informed consent was obtained prior to participation in the research. Within 2-weeks following completion of the exercise programs, consecutive participants were approached to conduct an interview via telephone or face-to-face at their outpatient clinic appointments. To allow for intervention maturation, recruitment of participants for qualitative interviews commenced after approximately the first 10% of participants had completed the intervention, and at times recruitment was dependent on staff availability to conduct the interviews. Figure 1 demonstrates participant recruitment and reasons for non-participation.

Table 1 provides a summary of the intervention in both the early- and late-commencing exercise studies and further details are published elsewhere[1, 5]. The interventions incorporated education in addition to mixed aerobic and resistance training in a group-based setting, supervised by a physiotherapist in a hospital environment. The early-commencing program started upon hospital admission, whilst the late-commencing program started at approximately 60-days post-transplant. Both interventions had a home-based, self-directed component and provided exercise tools such as a diary and/or activity monitoring device to enhance compliance.

***Data collection and analysis***

Participant demographics, including baseline physical activity levels, and feasibility data, including percentage attendance at available sessions, were collected for this study. A semi-structured interview schedule (Supplementary File 2) was developed to gather participant feedback regarding the intervention, in particular acceptability, program expectations, prior experiences, and beliefs regarding timing, barriers and facilitators. The first two completed interviews were reviewed by study lead (SA), and verbal prompts were added to the interview guide to maximise depth. Following completion and transcription of the semi-structured interviews with outpatient exercise program participants, the interview schedule for the inpatient exercise program participants was altered slightly to gain perspectives on designing future exercise programs (Supplementary File 2). All interviews were conducted one-on-one with only the interviewer and participant present within a maximum of 4-weeks following program completion. Of the total 35 interviews, 32 were conducted face-to-face in a private room within the hospital outpatient department, and 3 were conducted via telephone. A pragmatic sample of 15-20 participants from each program was chosen to allow transferability to other contexts[21, 22]. Given interviews were relatively short in length (average length 7.2minutes, range 3.1 to 15minutes), to facilitate sufficiently rich data to answer the research aim, data from both the outpatient and inpatient studies were combined rather than analysed separately.

Interviews were recorded and transcribed verbatim (GS), then checked by a second independent researcher for accuracy (SA). Transcripts were non-identifiable, with participants given a number, and stored electronically. Transcripts were uploaded into NVivo software (released in March 2020; QSR International Pty Ltd.) to be independently coded by two researchers (SA, SMP). Transcripts were identified as pertaining to participants from the outpatient or inpatient program using ‘OP’ or ‘IP’, respectively. This was done to highlight if specific codes and subsequent themes were only relevant to one cohort. Participant review of transcripts did not occur as it was deemed inappropriate due to risk of mortality in this population and time elapsed from interview to analysis (2-5years) [21, 23].

An inductive, conventional approach to qualitative content analysis as described by Hsieh & Shannon (2005) was used to analyse the data [24, 25]. This approach to qualitative content analysis avoids use of preconceived categories or counting and fits within the naturalistic paradigm and may have similarities to ‘ethnographic content analysis’ and inductive approaches to thematic analysis[24, 25]. Each transcript was read from start to finish, and then line by line to derive codes that appeared to capture participants’ perspectives[24]. The researchers independently grouped initial codes into categories to develop major themes and sub-themes from the data[24]. One researcher (SA) led both inpatient and outpatient interventions hence was innately engaged in the research topic and with the participants. Therefore, the independent researchers met after coding the first five interviews to ensure understanding of the context, check that preliminary interpretation of data into codes appropriately represented participant perspectives, reduce risk of bias and maximise credibility within a naturalistic paradigm[24, 25]. The two researchers then met again to cross-check the categorisation of codes into final themes and sub-themes until consensus was reached[24]. A third researcher was available however not required as consensus was reached. This third researcher (CG) reviewed the final themes alongside extracts from interview transcripts to check that the emerging themes were representative of participant quotations.

**Supplementary File 2:** Semi-structured interview guides for (a) Outpatient study; (b) inpatient study

**a: Outpatient study semi-structured interview guide**

1. What were the reasons you agreed to participate in this program?
2. At what day (roughly) following your transplant did you have your first exercise class? Did you feel this was an appropriate time? Why/why not?
3. Were there any barriers (things that hindered) or facilitators (things that helped) to your involvement in this program and if so, what were these?
4. Did you have any expectations about any benefit or harm out of participation in this program before you started? And if so, what were they?
5. Now that the program has ended, were those expectations realised? Or what do you believe you have gained or lost from this program, if anything?
6. Do you have any other comments?

**b: Early-commencing Inpatient study semi-structured interview guide**

1. What were the reasons you agreed to participate in this exercise study / program?
2. The exercise program on the ward started roughly within the first week of your hospital stay. Did you feel this was a good time to start exercising? *(pause)* Why/why not?
3. Did you have any expectations about any benefit or harm out of participation in this program before you started? And if so, what were they?
4. (a) Was there anything that stopped you (or were there any barriers) from coming along to the exercise group?

(b) What were the things that helped (facilitators) you to come along to the exercise group on the ward?

1. Now that the program has ended, did it meet your expectations? *(pause)*
   - What do you believe you have gained or lost from this program, if anything?
2. How have you used skills gained from this program after you went home from hospital?
3. If you were designing an exercise program for people having a bone marrow transplant, what do you think the exercise program should include?
   - What advice would you give us that could help us to make sure you get the right amount of exercise at the right time?
4. *For the first few participants* – do you think your views about the exercise program have changed over the past 60 days?
5. Thank you for participating in this interview – is there anything else you would like to add about your experience of undertaking the exercise program?

**Supplementary File 3 – Supplementary Table S1:** Major themes and sub-themes developed from participant interviews; examples of relevant participant quotations from interviews.

| **Major theme 1: Motivation, automatic or reflective, to exercise during or after BMT** | |
| --- | --- |
| **Sub-themes:**  Reflective motivation: belief that exercise would benefit recovery from transplant | IP13: “…good for my body, and exercise is good for the brain too. Good for… outlook on things, lots of benefits.”  OP43: “I wanted to make a good recovery and do everything I can to become the person I was before.” |
| Reflective motivation: belief that exercise would improve physical symptoms associated with treatment, in some cases based upon impact of prior experience of hospitalisation | IP21: “After my autograft, I was quite weak, which I didn’t have a physiotherapy program for, so I wanted to try and mitigate some weakness that having a transplant entails.”  OP21: “…it [exercise] was an opportunity…to rebuild some physical strength that I lost while I was in hospital.” |
| Reflective motivation: belief that participation in exercise would provide a sense of control, structure or distraction from treatment | IP07: “…it was a motivating factor, just to get up and move and it gets you out of the environment you’re in… Takes your mind off everything else that’s happening, and it’s a time when you tend to feel a little bit ordinary… the formality of having to go to a class helped as well.” |
| Automatic motivation: accountability to staff whom are trusted or believed to have knowledge and experience in BMT | OP39: “I just thought ah I don’t think I can do this. But the doctor… said look no I think it would be good, and so I did it.” |
| Automatic motivation: reinforcement to exercise through peer support in group setting or encouragement from staff | OP14: “I was still battling with my own personal motivation but towards the end of the class it just became so much easier, because each time you turned up you met up with the staff who have been fantastic and you met up with other BMTs and you got to talk to them, see where they’re going and try and encourage everyone so it was great and by doing that you encourage yourself as well to do more.” |
| Automatic motivation: behavioural regulation from exercise tools such as diary or ‘FitBit’ step-counting device | IP05: “…the diary where you can write it down and things like that is quite useful, and you can keep track of how often you’re doing it and what you’re doing.”  OP08: “having the Fitbit was amazing, able to track what you do and… we’d look at the Fitbit at night and only say 7,000 steps so we’d go for a walk and make it up to 10,000.” |
| Automatic motivation: from an ability to measure change | OP13: “So you can physically measure the outcome which is good.” |
| Automatic motivation: strong intrinsic motivation to exercise and continue this as part of lifestyle, often associated with prior exercise experience | IP21: “I did it because I was like ‘alright you have to do it…you feel sick doesn’t matter, you have to do it’.”  OP25: “I requested to start when I did because I was already exercising at home as it was. So, the more the better as far as I’m concerned.” |
| Automatic motivation: lack of intrinsic motivation, particularly for the unsupervised aspects of exercise | OP21: “The only thing I struggled with was doing the home exercises, at the beginning I was doing them quite frequently, but then I sort of lost motivation or got a bit lazy, sort of dropped off…” |
| **Major theme 2:** **Physical opportunity to exercise** | |
| **Sub-themes:**  Importance of access to equipment and suitable therapy space across inpatient, outpatient and home setting | IP26: “A better space, like we didn’t really have…we were in a meeting room, which is just part of being on the ward I guess, and sometimes we got booted out of there…”  IP12: “I think having access to the treadmills and bikes and stuff like that was great…outside of the sessions.” |
| Challenges of access to outpatient or home-based exercise: geographical, environmental/weather and logistical elements | OP13: “I mean the obvious one is trying to get here by 11am with the car-parking … so having those extra carpark passes that gives you double time that’s been a huge bonus for me.”  OP20: “…in the late periods of the program I was three hours interstate, … so it just didn’t work out.” |
| Managing time for exercise within busy lifestyle (at home) or appointment schedule (in hospital) | IP13: “Trying to coordinate everything, it was difficult to fit it in sometimes…”  OP42: “…I usually had other appointments on during the day so coming here was no issue.” |
| **Major theme 3: Capability to exercise** | |
| **Sub-themes:**  Impact of symptoms on physical capability to participate in an exercise program, particularly the severity of symptoms as a barrier in the inpatient setting | IP19: “I’d had a rough trot… I ended up in ICU… then I had a mouth full of ulcers and I just couldn’t operate I couldn’t do anything…”  OP30: “When you’re not feeling well obviously that makes you less enthusiastic about doing things, and certainly at times illness has prevented me from attending…” |
| Impact of fatigue and/or weakness on participation in exercise | IP29: “…I was so deconditioned that I was unable to attend the classes and had to have some special… assistance…I really struggled with sleep and fatigue…”  OP22: “Well a barrier is fatigue, being constantly fatigued, even now… I’m just feeling fatigued, so that’s been a barrier for me.” |
| Fluctuations in health status as a barrier to physical capability | OP43: “It was mainly me and my body itself, …low blood pressure … or I hadn’t eaten enough, I’m just not feeling well, or it’s too early in the morning…”  OP21: “…depending on how I felt physically, like some days I was a bit sick so I wasn’t able to perform as well, or some of the medication I’ve been on made me feel nauseous during the program. That was the only thing that blocked me.” |
| Psychological capability – varied beliefs, knowledge, value and confidence to exercise | IP12: “I’d done a bit of my own research so I had a level of understanding that if I could maintain some level of fitness throughout the process that it would make my recovery a whole heap better and also help me cope better with the transplant itself.”  OP41: “I actually initially thought it was way too soon because I was still not really walking very well or the rest, but once I got going it was, helped me get over that.” |
| **Major theme 4: Psychosocial effects of group-based exercise** | |
| **Sub-themes:**  Peer support and strong sense of community | IP06: “…the degree of socialization you got, I wouldn’t totally dismiss … you may come across other patients when you’re walking around the ward, but actually being in a room and talking to them… a bit of camaraderie, but also learning more about people that are ahead of you and seeing progress and things like that…”  OP21: “The other benefit that I found was developing relationships with some of the other patients, who are also going through the same thing, that was really helpful, sort of forming that community.” |
| Exercise, particularly in a group setting, supported mental health through social interaction in an otherwise isolating healthcare environment | IP21: “It was nice to get out of the room and talk with other people.”  OP41: “as a group, we’ve gelled really well… encouraging… and people at different stages who can help advise and support people at lower stages and… that hasn’t happened anywhere else in anything else I’m doing here at the hospital…” |
| Accountability or motivation from the group-based setting – gaining perspective from seeing others exercise | IP08: “Every time I see the group, even if they have many instruments in their body they are willing to do the exercise and they knew and believed that this will help them, and that… gives me… encouragement.”  OP43: “I was especially motivated by certain people who were really active and it was like nothing happened to them and I was like I wanna be like that as well. So, they inspired me actually to do exercises and to come down here and keep coming.” |
| Reluctance toward group-based exercise in some inpatient participants due to concerns regarding infection risk or that emotional investment in others would affect own progress. | IP09: “although they wipe down the equipment and stuff like that, there was still a lot of passing around of dumbbells… so I was a little bit more standoffish.”  IP22: “being quite an empathetic person, I just didn't wanna… get influenced by that and potentially affect my positive outlook. So that's why I stayed away.” |
| **Major theme 5: Experienced impact of participation in an exercise program** | |
| **Sub-themes:**  Experienced physical improvements including strength and/or symptom management | IP10: “I’m stronger now than what I was before the exercise…”  OP32: “It help a lot… like more energy and the short breath this is helping as well.” |
| Experienced benefit greater than or faster than expected post-transplant, which participants felt may be attributed to exercise participation | OP08: “…I felt that I recovered quicker than what I thought and I’m sure that the exercises helped.” |
| Some reported mismatch between expectation and reality with recovery progress | OP39: “I’m not as strong as I’d like to be, and actually I’d like to have been able to do exercise lately…” |
| Experienced benefits to emotional wellbeing or self-confidence | IP06: “I just felt better in my mind for participating each day, it felt like I’d done something.”  OP20: “…building the confidence again in the fact that your body is capable to actually do those things and sets you in the right mindset and the motivation train to go on and continue post treatment...” |
| Experienced a return to ‘normal’ life and moving in the right direction with recovery | IP03: “I think I’m back on track even going back to my normality of things at home…”  OP07: “the program helped a lot with being active, building up the activities that you want to do again which after the transplant I wasn’t able to do a lot of stuff and following the program… sort of put me back in to that routine again…” |
| Gained knowledge or skill development to continue exercising post program | OP27: “I gained a lot as in, I know how to do the exercises properly, I’ve been shown, guided through them very well the whole process has been good.” |
| **Major theme 6: Intervention design considerations** | |
| **Sub-themes:**  Importance of tailored exercise prescription based on symptoms, comorbidities, age, and interests | IP12: “…you’ve got such a broad array of people with different underlying conditions and so on and so forth so I think it has to be flexible.”  OP36: “…it’s tailored to your individual capabilities too at the time… personalised…which I think is an excellent way to go.” |
| Importance of education as an incentive or to empower self-management of recovery and implementation of an active lifestyle | IP13: “…selling the fact that …people tend to feel better after exercising… that, the more one exercises whilst an inpatient, the better their outcome is likely to be, to me that seems like… one of the better motivations.” |
| Potential involvement of family to increase motivation or accountability to exercise | IP06: “…maybe at least one or two times encouraging the carers to come along… that way they’d be able to motivate and encourage the patients… if they don’t feel self-motivated enough to do the exercises themselves or just to get them there in the first instance.” |
| Desire to commence a formal exercise program earlier in both inpatient and outpatient settings | OP25: “Personally, I could have probably started a bit earlier; I requested to start when I did because I was already exercising at home as it was. So, the more the better as far as I’m concerned.” |
| Need for continuity of and greater access to exercise support over the first 100-days post-transplant | IP12: “…would have liked almost the classes to continue between being discharged and the day 60 just to help us, kind of, help boost that motivation again.” |
| Flexibility regarding timing of program delivery | IP06: “…mornings are a very busy time of the day on the ward… having said that if you push it towards the afternoon, whether that will demotivate people to participate I’m not sure.”  OP20: “…there’s flexibility with [physiotherapy staff] as well, willing to work around appointments…” |
| Importance of structured exercise to be embedded into routine care to recover from transplant | IP09: “…without the program people would struggle to get through to the other end of the bone marrow transplant, it’s very, it’s very important for everybody.” |

**IP and OP indicate participant from early commencing inpatient and late commencing outpatient exercise program respectively*
